# Habitat imaging radiomics of chest CT identifies noninfectious acute exacerbations in chronic obstructive pulmonary disease

**DOI:** 10.3389/fmed.2025.1719017

**Published:** 2026-01-05

**Authors:** Zhenxing Feng, Shuo Liang, Minghui Hua, Yafang Zheng, Jiwei Sun, Li Zhou, Yimeng Zhang, Boxin Li, Yi Li, Baozhen Ge, Hong Zhang, Daqiang Sun

**Affiliations:** 1 Department of Radiology, Tianjin Chest Hospital, Tianjin, China; 2 Department of Respiratory and Critical Care Medicine, Tianjin Chest Hospital, Tianjin, China; 3 School of Precision Instrument and Opto-Electronics Engineering, Tianjin University, Tianjin, China; 4 Key Laboratory of Opto-Electronics Information Technology of Ministry of Education, Tianjin University, Tianjin, China; 5 Department of Thoracic Surgery, Tianjin Chest Hospital, Tianjin, China

**Keywords:** habitat, radiomics, chronic obstructive pulmonary disease, acute exacerbation, CT

## Abstract

**Objective:**

Noninfectious acute exacerbations of chronic obstructive pulmonary disease (AECOPD) pose significant diagnostic challenges due to the lack of reliable biomarkers. This study aims to develop and validate a CT-based habitat imaging radiomic model for precise identification of noninfectious AECOPD.

**Methods:**

This retrospective study included 352 eligible chronic obstructive pulmonary disease (COPD) patients who received treatment at Tianjin Chest Hospital from January 2019 to December 2023. Among these patients (181 with noninfectious AECOPD, 171 with stable COPD), stratified randomization allocated cohorts to training (*n* = 211) and testing (*n* = 141) cohorts. Whole-lung CT scans were subjected to habitat mapping by voxel-wise K-means clustering, with radiomic features derived from habitat subregions and optimized using least absolute shrinkage and selection operator regression. Logistic regression (LR) and support vector machine (SVM) models combined habitat-derived traits with clinical factors.

**Results:**

The CT-based whole lung was segmented into three habitat subregions: habitat subregion 1 (emphysema/bullae-associated), habitat subregion 2 (bronchovascular bundle), and habitat subregion 3 (lung parenchyma). The habitat_total_ model showed predictive power for identifying noninfectious AECOPD (training: AUC = 0.853 [LR], 0.897 [SVM]; test: AUC = 0.800 [LR], 0.807 [SVM]). Multivariate analysis identified habitat_total_ score and GOLD stage as independent predictors of noninfectious AECOPD (*p* < 0.001).

**Conclusion:**

In conclusion, this study segmented whole-lung CT scans of noninfectious AECOPD patients into habitat subregions, developing a radiomics model that demonstrated strong diagnostic efficacy. This approach provides an objective imaging biomarker and a potential tool for quantifying COPD heterogeneity.

## 1 Introduction

Chronic obstructive pulmonary disease (COPD) is a prevalent respiratory condition characterized by persistent airflow limitation and chronic inflammation, exhibiting significant pathophysiological heterogeneity (1, 2). Acute exacerbations of COPD (AECOPD) are critical clinical events that accelerate lung function decline, increase hospitalization rates, and severely impair quality of life (3, 4). Although approximately 80% of AECOPD cases are of infectious origin-often identifiable by radiographic infiltrates on computed tomography (CT) (5) and elevated inflammatory biomarkers such as leukocytosis and C-reactive protein -noninfectious exacerbations triggered by environmental factors (e.g., tobacco smoke, air pollutants, and inhaled allergens) represent a distinct clinical entity (6).

The precise identification of noninfectious AECOPD remains a formidable clinical challenge due to several fundamental limitations. First, the absence of objective biomarkers forces reliance on subjective symptom evaluation. In stable COPD patients, chronic respiratory symptoms (e.g., dyspnea, cough, sputum production) often mask acute deteriorations, leading to under-recognition of noninfectious exacerbations (7). Second, conventional diagnostic tools exhibit insufficient sensitivity. While traditional CT metrics and inflammatory biomarkers effectively identify infectious etiologies, they lack relevance for noninfectious cases, where such parameters typically remain unchanged (8–10). Third, comorbid cardiopulmonary conditions frequently mimic or exacerbate AECOPD symptoms, further complicating differential diagnosis (6).

This diagnostic gap has direct therapeutic implications. Misclassification of noninfectious AECOPD contributes significantly to antibiotic overuse, fueling bacterial resistance and underscoring the urgent need for precise phenotyping tools (11, 12). Consequently, there is a pressing demand for advanced diagnostic strategies capable of capturing the subtle and heterogeneous pathophysiological changes associated with noninfectious exacerbations, which are not discernible through conventional CT imaging or biomarker assays.

To address this unmet need, we turned to habitat imaging radiomics-an innovative analytical framework that transcends conventional whole-lesion analysis by mapping intrapulmonary spatial heterogeneity at the voxel level. Originally developed in oncology to characterize tumor subregions (13–16).

In this study, we hypothesize that CT-based habitat imaging can delineate distinct parenchymal and airway subregions in COPD, capturing pathophysiological patterns specific to noninfectious AECOPD. By deriving and validating a habitat radiomics model, we aim to provide a novel, non-invasive tool for the accurate identification of noninfectious exacerbations, thereby facilitating targeted therapeutic interventions and advancing personalized management in COPD.

## 2 Materials and methods

### 2.1 Patients and study design

A retrospective analysis was performed with 1,261 COPD patients treated at Tianjin Chest Hospital from January 2019 to December 2023. Table 1 and Supplementary Table S1 summarize cohort demographics, smoking history, inflammatory markers, pulmonary function parameters, and comorbidities. Non-contrast chest CT scans were conducted for all patients. The Ethics Committee of the Tianjin Chest Hospital granted stringent clearance for this investigation (Approval No. 2024KY-025-01).

**Table 1:** Comparative baseline characteristics by disease status and cohort.

Clinical features	Training cohort (*n* = 211)			Test cohort (*n* = 141)		
	Stable COPD (*n* = 102)	AECOPD (*n* = 109)	*p-*value	Stable COPD (*n* = 69)	AECOPD (*n* = 72)	*p*-value
Age			<0.001			0.023
<68 years	71(69.61%)	47(43.12%)		42(60.87%)	29(40.28%)	
≥68 years	31(30.39%)	62(56.88%)		27(39.13%)	43(59.72%)	
Gender			0.387			0.293
Male	87(85.29%)	87(79.82%)		61(88.41%)	58(80.56%)	
Female	15(14.71%)	22(20.18%)		8(11.59%)	14(19.44%)	
Smoking History			0.901			0.053
Smoker	43(42.16%)	44(40.37%)		32(46.38%)	21(29.17%)	
Never	59(57.84%)	65(59.63%)		37(53.62%)	51(70.83%)	
GOLD stage			<0.001			<0.001
GOLD I-II	76(74.51%)	39(35.78%)		45(65.22%)	23(31.94%)	
GOLD III-IV	26(25.49%)	70(64.22%)		24(34.78%)	49(68.06%)	
FEV1 (L)	1.70 ± 0.67	1.27 ± 0.65	<0.001	1.61 ± 0.67	1.27 ± 0.53	0.002
FVC (L)	2.96 ± 0.82	2.46 ± 0.83	<0.001	2.85 ± 0.81	2.44 ± 0.68	0.001
FEV1/FVC%	54.76 ± 10.94	49.07 ± 11.87	<0.001	55.02 ± 12.70	50.68 ± 10.90	0.008
FEV1%pred	61.38 ± 18.04	45.93 ± 20.74	<0.001	58.17 ± 21.01	47.89 ± 17.56	0.003
WBC (×10^9^/L)	6.21 ± 1.81	6.35 ± 1.68	0.522	6.73 ± 1.68	6.70 ± 1.59	0.880
NEUT%	56.64 ± 10.30	57.64 ± 9.73	0.474	57.61 ± 10.52	56.84 ± 10.60	0.782
CRP (mg/L)	3.80 ± 2.36	4.03 ± 2.22	0.473	4.03 ± 2.41	3.85 ± 2.42	0.782
CVD			0.284			1.000
Yes	30(29.41%)	24(22.02%)		13(18.84%)	13(18.06%)	
No	72(70.59%)	85(77.98%)		56(81.16%)	59(81.94%)	

FEV1, Forced Expiratory Volume in 1 s; FVC, Forced Vital Capacity; FEV1%pred, FEV1 percent predicted; WBC, White Blood Cell count; NEUT%, Neutrophil percentage; CRP, C-reactive protein; CVD, Cardiovascular Disease.

Noninfectious AECOPD was defined per the GOLD 2025 criteria (17) as an acute worsening of respiratory symptoms (dyspnea, cough, or sputum production) occurring within 14 days, with an increase beyond normal day-to-day variation. Infectious AECOPD was rigorously excluded based on comprehensive assessment including: (1) the absence of new parenchymal infiltrates on chest CT scans as evaluated by two independent radiologists; (2) normal inflammatory markers (white blood cell count < 10 × 10^9^/L and C-reactive protein < 8 mg/L); and (3) no clinical evidence of respiratory infection as determined by the treating physician. Supplementary Figure S1 illustrates representative CT images from patients with stable COPD and noninfectious AECOPD. The visual evaluation revealed little inter-phenotype variations, requiring quantitative biomarker investigation. This stringent definition ensured a pure cohort for investigating the distinct pathophysiological characteristics of noninfectious exacerbations.

The inclusion criteria were: (1) completion of pulmonary function tests and non-contrast CT within 7 days; (2) thorough clinical documentation. Other criteria for exclusion included: (1) Inferior CT quality (motion artifacts or reconstruction thickness exceeding 2 mm); (2) thoracic comorbidities such as pleural effusion, pulmonary atelectasis, or lung nodules greater than 5 mm; (3) pulmonary neoplasms; (4) previous thoracic surgery. Patients were randomly divided into training (*n* = 211) and test (*n* = 141) cohorts in a 6:4 ratio. Supplementary Figure S2 illustrates the screening cascade.

### 2.2 CT acquisition protocol

Inspiratory-phase non-contrast CT scans were obtained in the supine position using scanners from two vendors: Siemens SOMATOM Force (Erlangen, Germany) or Philips Brilliance iCT 256 (Best, Netherlands). Standardized parameters included 120 kV tube voltage, tube current modulation (200–300 mA range), and a reconstructed matrix size of 512 × 512. The scan field of view (SFOV) was adjusted to patient anatomy, resulting in an in-plane pixel spacing ranging from approximately 0.60 mm to 0.75 mm. The slice thickness was 1.5 mm reconstructed at 1.5 mm intervals.

To minimize the influence of inter-scanner variability and anisotropic voxel dimensions on radiomic feature extraction-a critical consideration for ensuring model robustness-all DICOM images were resampled to a standardized space of 1 mm^3^ isotropic voxels using linear interpolation prior to habitat mapping and feature extraction (18).

### 2.3 Image processing and whole-lung habitat mapping

To ensure segmentation stability and reproducibility, the whole-lung regions of interest (ROIs) were automatically delineated using the TotalSegmentator extension (Revision: 76aaa53) implemented in the 3D Slicer platform (v5.6.2). This publicly available tool has been extensively validated and demonstrates robust, highly reproducible segmentation of anatomical structures on CT imaging (19). The resulting segmentations were subsequently reviewed and, when necessary, manually adjusted by two board-certified radiologists, each with over 10 years of specialized experience in thoracic imaging. This combined automated and expert-driven approach provided a consistent and reliable foundation for subsequent habitat mapping and radiomic feature extraction (Supplementary Figure S3).

K-means clustering was applied at the voxel level using Python 3.12, with clustering based exclusively on the normalized CT intensity values to capture tissue density variations. The cluster number k was varied from 2 to 9, a range selected based on common practices in habitat imaging literature to explore a reasonable number of clusters without over-segmentation, while balancing computational feasibility and biological interpretability. The optimal k was identified globally for the entire cohort by maximizing the Calinski-Harabasz index (20), and its stability was validated through 1,000 bootstrap iterations to ensure robustness (21, 22).

### 2.4 Radiomic feature extraction, modeling, and validation

Radiomic features were extracted from the three habitat subregions to develop clinically actionable biomarkers. All CT images underwent a standardized preprocessing pipeline to ensure consistency. For texture calculation, gray-levels were discretized using a fixed bin width of 5 Hounsfield Units (HU).

Using the PyRadiomics library (v3.0.1) in Python, a comprehensive set of 1,834 features was extracted from each habitat subregion across the entire lung volume. This resulted in a pool of 1,834 features per subregion, which included first-order statistics, texture attributes derived from the Gray-Level Co-occurrence Matrix (GLCM), Gray-Level Dependence Matrix (GLDM), Gray-Level Run-Length Matrix (GLRLM), Gray-Level Size Zone Matrix (GLSZM), and Neighboring Gray-Tone Difference Matrix (NGTDM), along with three-dimensional morphological features (23, 24). The features extracted from each subregion were aggregated per subject, collectively forming a global radiomic signature for each habitat type, which served as the input for subsequent modeling.

Prior to modeling, the feature dataset underwent a multi-step preparation and selection process. All features were normalized using Z-score standardization. To mitigate redundancy and address potential multicollinearity prior to the primary feature selection, we first calculated pairwise Pearson correlations (|*r*| > 0.9) among all features. Within each highly correlated pair, only the feature demonstrating the stronger univariate association with the outcome (noninfectious AECOPD) was retained for subsequent analysis. This was followed by univariate screening to identify features with significant differences between noninfectious AECOPD and stable COPD groups using independent t-tests or Mann–Whitney U tests, preserving variables with *p* < 0.05.

The final stage of feature selection employed Least Absolute Shrinkage and Selection Operator (LASSO) regularization with 10-fold cross-validation. The optimal lambda (*λ*) value, which minimizes the binomial deviance, was selected to shrink the coefficients of non-contributory features to zero, resulting in a parsimonious and optimal subset of predictors for model construction.

Diagnostic models were constructed using L2-penalized logistic regression (LR) and radial basis function support vector machine (SVM), trained on the optimized feature set. Diagnostic models employed L2-penalized logistic regression for interpretability and probability outputs, and SVM for high-dimensional non-linear radiomics, balancing clinical applicability with predictive power (24). Validation of the test cohort utilized receiver operating characteristic (ROC) analysis, with the area under the curve (AUC) assessing diagnostic discrimination. A clinical-radiomic integrated nomogram was created to illustrate coupled predictors. Prediction calibration was assessed using calibration curves, and clinical net benefit across 5–95% risk thresholds was evaluated by decision curve analysis utilizing the devtools package. Supplementary Figure S3 illustrates the comprehensive analytical process.

### 2.5 Statistical analysis

Statistical analyses were conducted using GraphPad Prism 10 (San Diego, CA), R version 4.4.2, and Python version 3.12. The measurement of normality utilized the Shapiro–Wilk test. Parametric continuous data are presented as mean±SD and subjected to independent t-testing; nonparametric data were transformed to median (IQR) and analyzed using the Mann–Whitney U test. Categorical variables exhibited frequencies and were subjected to x^2^ testing. The discriminative efficacy of noninfectious AECOPD was assessed using ROC-derived AUCs, and model comparisons were conducted employing DeLong’s technique.

## 3 Results

### 3.1 Patient demographics and baseline characteristics

This study included 352 COPD patients, predominantly male (241 males, 111 females), with a median age of 68 years. The group comprised 181 noninfectious AECOPD patients and 171 stable COPD controls. Comprehensive baseline characteristics are detailed in Supplementary Table S1. Patients were assigned to training (*n* = 211) and test (*n* = 141) cohorts via stratified randomization. Equivalence testing validated the balanced distributions of age, sex, smoking status, GOLD stage, and AECOPD prevalence between the training and test cohorts (all *p* > 0.05; Supplementary Table S2), hence affirming cohort comparability for subsequent modeling.

The comparative analysis revealed that noninfectious AECOPD patients were markedly older than stable COPD controls in both the training and test cohorts (all *p* < 0.05). Significantly impaired lung function and elevated GOLD stages characterized noninfectious AECOPD groups in both cohorts (*p* < 0.05). The distribution of sex and smoking status exhibited no significant variations between illness stages (all *p* > 0.05). The results are presented in Table 1.

### 3.2 Habitat imaging and radiomic model development

**Figure 1:**
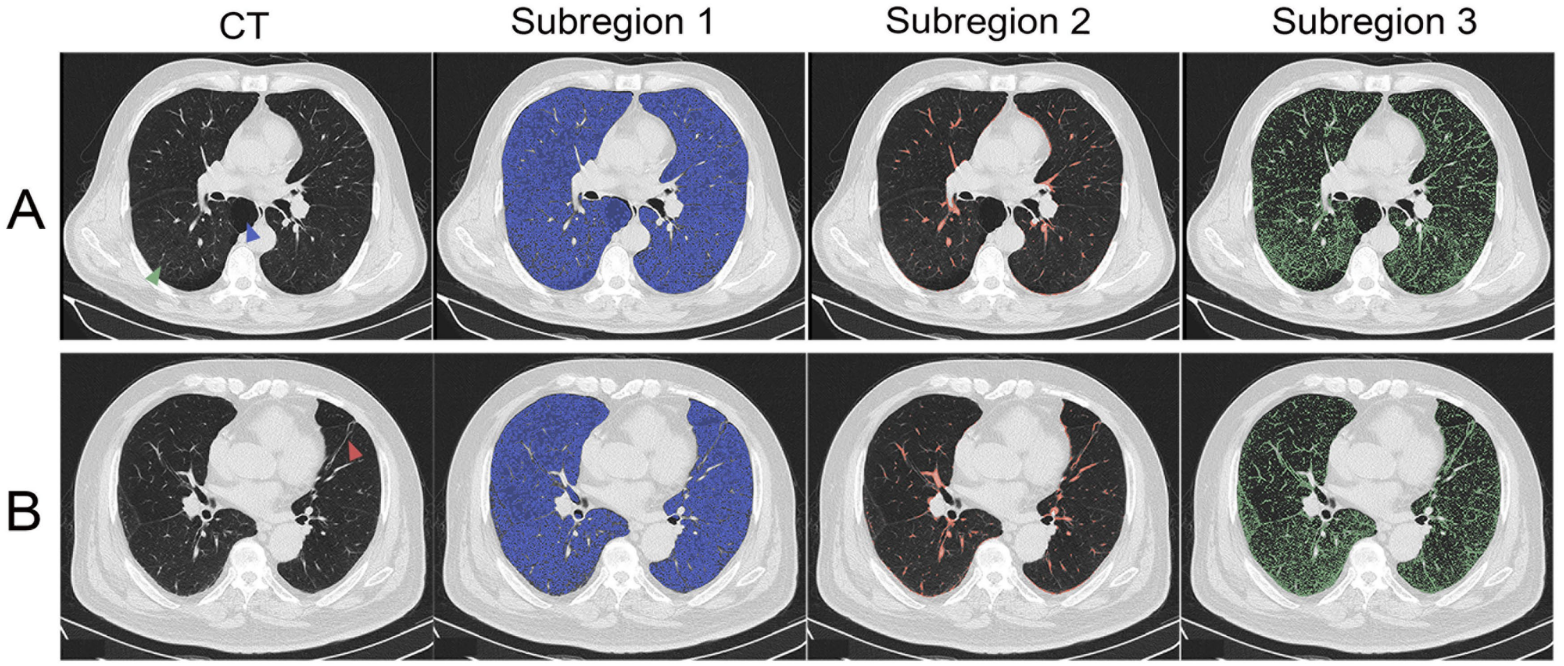
Display of habitat subregions 1–3 in different COPD patients. **(A)** A 69-year-old man with noninfectious AECOPD. **(B)** A 75-year-old man with stable COPD. The lung parenchyma was partitioned into three distinct subregions: subregion 1 (blue) predominantly corresponded to areas of emphysema and bullae; subregion 2 (red) predominantly corresponded to bronchovascular bundles; and subregion 3 (green) predominantly corresponded to functional lung parenchyma. The bulla is classified as subregion 1 (blue arrow). Strip-like fibrotic foci along the bronchovascular bundles are classified as subregion 2 (red arrow). Ground-glass opacity is classified as subregion 3 (green arrow).

Habitat imaging analysis (Supplementary Figure S4) indicated that the Calinski-Harabasz score reached its maximum value of 3.15 × 10^4^ when the optimal number of clusters for the entire lung was three. Consequently, the whole lung parenchyma was segmented into three distinct habitat subregions. Habitat subregion 1 primarily correlated with emphysema and bullae, habitat subregion 2 predominantly encompassed the bronchovascular bundle (the CT-visible complex of airways, pulmonary vessels, and peribronchovascular interstitium), and habitat subregion 3 corresponded to lung parenchyma. Figure 1 clearly demonstrates this correspondence in representative patients: a 69-year-old man with noninfectious AECOPD (Figure 1A) and a 75-year-old man with stable COPD (Figure 1B), where bullae, bronchovascular bundles, and parenchymal changes are distinctly partitioned into the respective subregions. Supplementary Figure S5 illustrates the whole-lung distribution of the three subregions in a representative patient, further confirming the anatomical consistency and stability of this partitioning approach.

Due to the diffuse and diverse clinical involvement in COPD, radiomic characteristics were computationally amalgamated into a comprehensive whole-lung habitat signature (habitat_total_) encompassing all subregions (Supplementary Figure S6A). Following the screening for AECOPD relevance (Supplementary Figure S6B), LASSO selection identified 11 critical characteristics for the habitat_total_ model (Supplementary Figure S7), with 4, 12, and 9 features selected for habitat subregion models 1-3(Supplementary Figure S8), respectively. The distribution of the 11 features comprising the habitat total model across the three habitat subregions: subregion 1 contributed 3 features; subregion 2 contributed 4 features; and subregion 3 contributed 4 features. Overall, textural features predominated (10/11), with first-order features being less frequent (1/11), indicating that structural heterogeneity is the primary driver of the model’s predictive capability for noninfectious AECOPD. For comparison, traditional radiomic features were also extracted from the whole-lung volume without habitat segmentation. This model is referred to as “Rad_total_ model. LASSO selection identified 10 core features for the traditional Rad_total_ model (Supplementary Figure S9).

**Figure 2:**
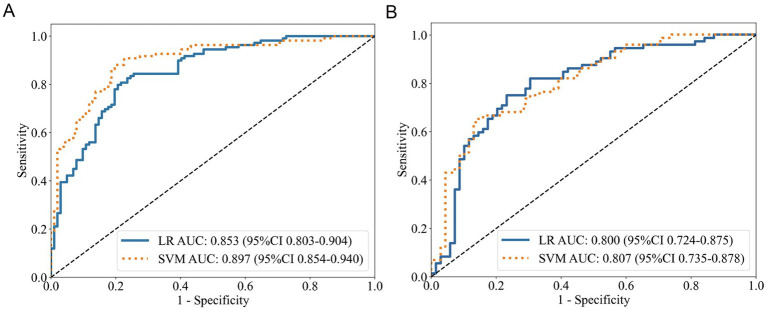
ROC curves of the habitat_total_ model. **(A)** Training cohort performance. **(B)** Test cohort validation.

Logistic regression analysis indicated that in the training cohort (Table 2; Figure 2A), the habitat_total_ model exhibited enhanced discriminative performance (AUC = 0.853) compared to subregion-specific models (subregion 1: 0.785; subregion 2: 0.796; subregion 3: 0.783). The advantage remained in the test cohort (Table 3; Figure 2B), with the habitat_total_ model attaining an AUC of 0.800 (subregion 1: 0.767; subregion 1: 0.692; subregion 1: 0.727). Probability histograms (Supplementary Figure S10) exhibited strong discrimination without overfitting, characterized by: (1) Gradual probability transitions from negative to positive classifications, and (2) Uniform distribution profiles across training and test cohorts. The SVM study further validated the superiority of the habitat_total_ model (training AUC = 0.897, test AUC = 0.807), designating it as the most effective predictor for subsequent applications.

**Table 2:** Diagnostic performance of habitat models in training cohort.

LR model	AUC	95%CI	Sensitivity	Specificity	Accuracy
Habitat_total_	0.853	0.802–0.903	0.789	0.794	0.791
Rad_total_	0.829	0.774–0.884	0.734	0.804	0.768
Subregion 1	0.785	0.723–0.845	0.706	0.725	0.716
Subregion 2	0.796	0.736–0.855	0.697	0.765	0.73
Subregion 3	0.783	0.721–0.844	0.771	0.676	0.725
SVM model					
Habitat_total_	0.897	0.854–0.939	0.872	0.804	0.839
Rad_total_	0.894	0.849–0.938	0.835	0.824	0.829
Subregion 1	0.805	0.746–0.864	0.697	0.784	0.739
Subregion 2	0.869	0.821–0.917	0.725	0.873	0.796
Subregion 3	0.842	0.789–0.894	0.780	0.765	0.773

AUC, area under curve; LR, Logistic Regression; SVM, Support Vector Machine; CI, Confidence Interval.

**Table 3:** Diagnostic performance of habitat models in test cohort.

LR model	AUC	95%CI	Sensitivity	Specificity	Accuracy
Habitat_total_	0.800	0.724–0.875	0.736	0.768	0.752
Rad_total_	0.769	0.690–0.847	0.667	0.768	0.716
Subregion 1	0.767	0.689–0.844	0.806	0.623	0.716
Subregion 2	0.692	0.603–0.780	0.681	0.696	0.688
Subregion 3	0.727	0.644–0.809	0.486	0.870	0.674
SVM model					
Habitat_total_	0.807	0.735–0.878	0.625	0.870	0.745
Rad_total_	0.757	0.677–0.836	0.764	0.652	0.709
Subregion 1	0.775	0.699–0.850	0.847	0.551	0.702
Subregion 2	0.746	0.664–0.828	0.778	0.652	0.716
Subregion 3	0.717	0.631–0.802	0.847	0.522	0.688

AUC, area under curve; LR, Logistic Regression; SVM, Support Vector Machine; CI, Confidence Interval.

**Figure 3:**
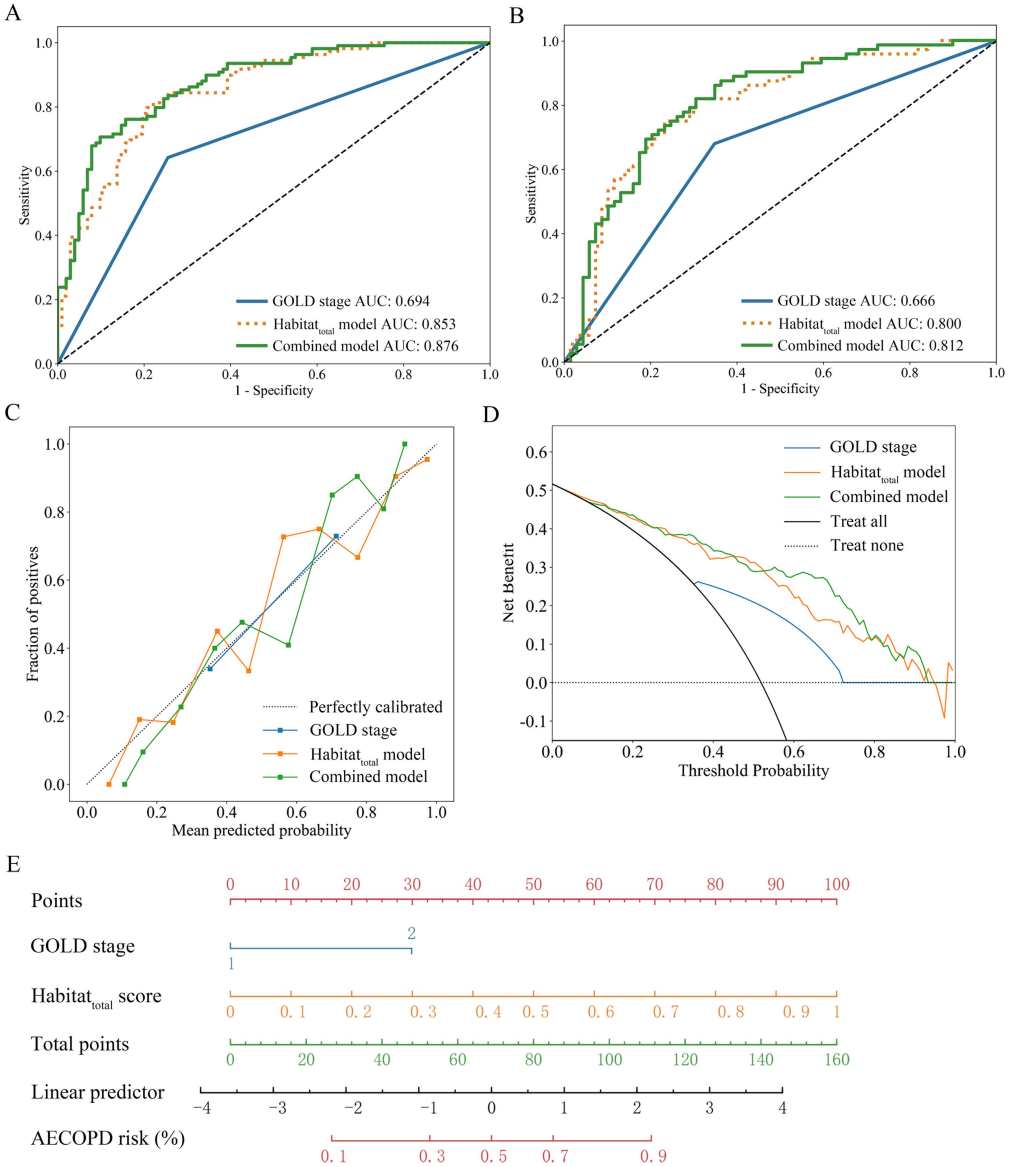
Diagnostic performance and clinical utility of the integrated model. **(A)** ROC curves in training cohort. **(B)** ROC curves in test cohort. **(C)** Calibration curve demonstrating prediction accuracy. **(D)** Decision curve analysis showing net clinical benefit. **(E)** Clinical nomogram integrating habitat_total_ model and GOLD stage.

Compared to the Rad_total_ model, the Habitat_total_ model achieved higher AUC values in both the training and test sets for both LR and SVM analyses (Tables 2, 3). However, DeLong’s test indicated that these differences were not statistically significant (all *p* > 0.05). As shown in Figures 3A,B and Supplementary Figure S11, the habitat_total_ model demonstrated significantly superior performance compared to GOLD staging alone in both the training and test cohorts (Training cohort: habitat_total_ AUC = 0.853 vs. GOLD stage AUC = 0.694, *p* < 0.001; Test cohort: habitat_total_ AUC = 0.800 vs. GOLD stage AUC = 0.666, *p* < 0.05).

### 3.3 Integrated diagnostic model development

Logistic regression established GOLD stage as a significant predictor of noninfectious AECOPD in both univariate and multivariate analysis across training and test cohorts (all *p* < 0.05, Table 4). The habitat_total_ score consistently exhibited strong predictive capability across all analyses (all *p* < 0.001). Age demonstrated notable correlations in univariate analysis (training *p* = 0.021, test *p* = 0.038) but not in multivariate analysis (both *p* > 0.05). The smoking history was significant only in the test cohort (*p* = 0.036). The findings identify the GOLD stage and habitat_total_ score as independent predictors of non-infectious AECOPD.

**Table 4:** Multivariate logistic regression analysis of noninfectious AECOPD predictors.

Training cohort	Univariate analysis		Multivariate analysis	
	OR(95%CI)	*p-*value	OR (95%CI)	*p-*value
Age	1.107 (0.540–1.674)	<0.001	0.733 (−0.015–1.460)	0.055
Gender	0.386 (−0.339–1.114)	0.297	1.00 (Reference)	
Smoking history	0.074 (−0.219–0.373)	0.792	1.00 (Reference)	
GOLD stage	1.658 (1.060–2.228)	<0.001	1.558 (0.798–2.318)	<0.001
Habitat_total_ score	5.556 (3.791–7.422)	<0.001	5.214 (3.683–6.756)	<0.001
Test cohort				
Age	0.833 (0.159–1.507)	0.015	0.458 (−0.387–1.303)	0.279
Gender	0.612 (−0.328–1.560)	0.203	1.00 (Reference)	
Smoking history	0.741 (0.047–1.435)	0.036	0.916 (0.187–1.645)	0.036
GOLD stage	1.386 (0.682–2.088)	<0.001	0.817 (0.007–1.627)	0.049
Habitat_total_ score	4.088 (2.674–5.502)	<0.001	3.661 (2.114–5.208)	<0.001

OR, odds ratio; CI, confidence interval. OR values were log-transformed.

A diagnostic model integrating GOLD stage and habitat_total_ score was established by logistic regression, with representation via a nomogram. The integrated model attained AUCs of 0.876 (95% CI 0.830–0.922) and 0.812 (95% CI 0.738–0.884) for AECOPD detection in the training and test cohorts, respectively (Figures 3A,B), surpassing the performance of standalone GOLD staging (training AUC 0.694, 95% CI 0.632–0.756; test AUC 0.666, 95% CI 0.588–0.745).

The calibration curves demonstrated a high level of predictive accuracy for the integrated model, habitat_total_ model, and GOLD stage (Figure 3C). Decision curve analysis demonstrated a greater clinical net benefit for the integrated model compared to comparator methods across various risk thresholds (Figure 3D). DeLong’s test (Supplementary Figure S11) demonstrated significantly elevated AUCs for both habitat_total_ and integrated models compared to GOLD staging (all *p* < 0.05), while revealing no significant AUC difference between habitat_total_ and integrated models (both *p* > 0.05). Figure 3E illustrates the clinical implementation nomogram.

Supplementary Figure S12 illustrates that the habitat_total_ score was markedly increased in advanced COPD severity groups (GOLD III + IV) relative to mild–moderate groups (GOLD I + II) in both training and test cohorts (all *p* < 0.001).

## 4 Discussion

Beyond oncology, habitat imaging research remains limited. Among the few existing studies, Yu Gao et al. pioneered its application in cerebrovascular disease (25). To our knowledge, this represents the first application of oncology-inspired habitat imaging to COPD exacerbation phenotyping, further extending its utility beyond tumor heterogeneity assessment. The pathological heterogeneity of COPD is evident in the many symptoms of airway remodeling, which include smooth muscle hypertrophy, fibrotic modifications, emphysematous destruction, inflammatory infiltration, and vascular abnormalities, among patients categorized purely by airflow limitation (26). Existing diagnostic frameworks inadequately define these multifaceted processes. The lack of dependable biomarkers for noninfectious AECOPD illustrates this intrinsic biological complexity (11, 12, 27). CT offers a noninvasive method for assessing structural pathology, whereas radiomics facilitates the computational extraction of subvisual characteristics to objectively evaluate disease heterogeneity (28). In this study, chest CT served dual roles: primarily excluding infectious etiologies of AECOPD, and secondly enabling habitat imaging analysis to characterize quantitative heterogeneity in noninfectious exacerbations.

Habitat imaging is an emerging analytical framework that delineates different radiomic subregions at the voxel level, providing new insights into pathophysiologic heterogeneity (13). In our study, the optimal habitat subregions (k = 3) identified through data-driven clustering correspond to distinct anatomical and pathological patterns in AECOPD, as supported by visual analysis. Importantly, the three habitat subregions offer a novel framework for interpreting COPD heterogeneity. Specifically:

- Habitat subregion 1, characterized by very low attenuation, corresponds to emphysematous destruction, which impairs compensatory ventilation during exacerbations.
- Habitat subregion 2, localized to the bronchovascular bundle, likely reflects airway-centric inflammation and remodeling, a key driver of exacerbations.
- Habitat subregion 3, representing the functional parenchyma, may exhibit textural changes indicative of diffuse parenchymal inflammation or micro-structural alterations that are not visually apparent on CT.

Thus, the habitat radiomics model quantifies the spatial distribution of these distinct pathological processes, enabling more precise identification of non-infectious AECOPD. Collectively, these findings demonstrate that the habitat subregions represent a novel imaging biomarker capable of capturing the underlying structural and functional heterogeneity in COPD.

Furthermore, segmenting the whole lung into these three distinct habitats aligns with the conventional diagnostic approach employed by thoracic radiologists when interpreting CT scans of COPD patients. Radiologists routinely evaluate disease heterogeneity by separately assessing components of emphysematous destruction, airway abnormalities, and parenchymal changes. Our data-driven habitat segmentation mirrors this clinical reasoning process, thereby enhancing the biological plausibility and translational potential of our radiomic model.

In this study, the habitat radiomics model (AUCs: 0.800/0.897) marginally outperformed conventional radiomics and exceeded the AUC of 0.76 reported in a recent Chinese AECOPD study (29). The tendency for higher AUC in the habitat-based model can be explained by its fundamental design. Habitat segmentation deconstructs the lung into physiologically distinct subregions, allowing radiomic features to more precisely reflect local pathological changes. In contrast, the traditional whole-lung ROI approach homogenizes this quantitative heterogeneity, potentially averaging out critical diagnostic information present in specific compartments.

Radiomic analysis across three habitat subregions reveals distinct pathophysiological mechanisms in COPD. Habitat subregion 1 (emphysema) features first-order intensity markers (e.g., low-attenuation indicators) reflecting alveolar destruction and tissue density loss (28, 30). Habitat subregion 2 (bronchovascular bundle) exhibits texture heterogeneity associated with airway wall thickening and inflammatory remodeling (28, 30). Habitat subregion 3 (functional parenchyma) demonstrates specific texture patterns, particularly GLDZM and GLSZM features, which capture subtle parenchymal heterogeneity indicative of early microstructural damage and ventilation-perfusion imbalance (24).

Multivariate analysis confirmed both GOLD stage and habitat_total_ score as independent predictors of noninfectious AECOPD, with the habitat_total_ score demonstrating the strongest association (*p* < 0.001). Furthermore, the significantly higher diagnostic performance of the habitat_total_ model compared to GOLD staging suggests that habitat imaging captures critical aspects of pathophysiological heterogeneity in AECOPD that are not fully represented by the global assessment of airflow limitation. The GOLD stage, while a cornerstone for severity classification, primarily reflects integrated pulmonary function, exhibits difficulties in addressing heterogeneity (23). In contrast, the habitat radiomics features, derived from spatially resolved CT data, may quantify distinct regional processes such as parenchymal destruction, airway inflammation, and vascular remodeling, which are pivotal in acute exacerbations. This synergy between functional staging and imaging-based heterogeneity assessment provides a more comprehensive tool for phenotyping noninfectious AECOPD.

The strong correlation between habitat_total_ score and advanced GOLD stages further suggests its potential as a quantitative marker of disease progression. We integrated the habitat radiomics and GOLD stage into a combined model and nomogram. Both the combined model and the habitat_total_ model demonstrated consistently robust predictive performance across both training and test cohorts. The nomogram combining habitat_total_ score and GOLD stage serves as a practical tool for clinical decision-making. Its graphical interface further enables rapid identification of noninfectious AECOPD, enhancing point-of-care diagnostic precision.

This study has several limitations. A primary limitation is the exclusion of infectious AECOPD cohorts, potentially restricting the generalizability of our findings. Additionally, the diagnostic criteria for noninfectious AECOPD rely heavily on subjective clinical indicators, which may introduce observer-dependent bias and affect diagnostic accuracy. Second, although CT habitat imaging allowed quantitative analysis in AECOPD patients, the lack of histopathological specimens prevented direct validation of pulmonary tissue heterogeneity. Thus, the biological relevance of radiomic features to AECOPD pathogenesis remains unverified, limiting model interpretability.

Future directions should encompass a prospective, multi-center study with a larger cohort that includes patients with infectious AECOPD. This will allow for external validation of the habitat radiomics model, direct comparison of its efficacy in differentiating infectious and non-infectious exacerbations, and further verification of the heterogeneity of COPD habitat subregions and the biological interpretability of radiomic features.

## 5 Conclusion

In conclusion, this is the inaugural study utilizing oncology-derived habitat imaging for phenotyping COPD exacerbations. Two primary conclusions arise: CT-based habitat imaging allows for the practical division of COPD lungs into different visualized subregions, hence enabling quantitative assessment of pathophysiological heterogeneity. The habitat radiomics model exhibits promising diagnostic efficacy for the detection of noninfectious AECOPD. These advancements offer innovative insights for the early detection of exacerbations and furnish doctors with practical instruments for the precise management of COPD.

## Data Availability

The raw data supporting the conclusions of this article will be made available by the authors, without undue reservation.
