# Supplementary material for: Habitat imaging radiomics of chest CT identifies noninfectious acute exacerbations in chronic obstructive pulmonary disease
Source: Front Med (Lausanne). 2026 Jan 5;12:1719017. doi: 10.3389/fmed.2025.1719017 (PMC12812631; doi:10.3389/fmed.2025.1719017)
Supplement: Supplementary file 1 [file Data_Sheet_1.pdf]

Supplementary Table S1. Demographic and clinical characteristics of the study cohort.

Variables	Total (n=352)	AECOPD (n=181)	Stable COPD (n=171)	*p* value
Age (years)				0.001
Mean ± SD	65.2 ± 9.8	67.5 ± 8.9	62.9 ± 10.1	
Age				0.009
<68 years	172 (48.9%)	78 (43.1%)	94 (54.9%)	
≥68 years	180 (51.1%)	103 (56.9%)	77 (45.1%)	
Gender				0.267
Male	241 (68.5%)	123 (67.9%)	118 (69.0%)	
Female	111 (31.5%)	58 (32.1%)	53 (31.0%)	
Smoking history				0.042
Smoker	207 (58.8%)	112 (61.9%)	95 (55.6%)	
Never	145 (41.2%)	69 (38.1%)	76 (44.4%)	
GOLD stage				0.005
GOLD I-II	184 (52.3%)	85 (46.9%)	99 (57.9%)	
GOLD III-IV	168 (47.7%)	96 (53.1%)	72 (42.1%)	
FEV1 (L)	1.46 ± 0.67	1.27 ± 0.61	1.66±0.67	<.001
FVC (L)	2.68±0.83	2.45±0.77	2.92±0.82	<.001
FEV1/FVC%	52.21±11.83	49.71±11.49	54.86±11.64	<.001
FEV1%pred	53.21±20.50	46.71±19.51	60.08±19.30	<.001
WBC ($\times 10^9$/L)	6.46±1.71	6.49±1.65	6.42±1.77	0.679
NEUT%	57.18±10.20	57.32±10.06	57.03±10.37	0.791
CRP (mg/L)	3.93±2.33	3.96±2.30	3.89 ± 2.37	0.789
CVD				0.293
Yes	272 (77.27%)	128 (74.85%)	144 (79.56%)	
No	80 (22.73%)	43 (25.15%)	37 (20.44%)	

FEV1, Forced Expiratory Volume in 1 second; FVC, Forced Vital Capacity; FEV1%pred, FEV1 percent predicted; WBC, White Blood Cell count; NEUT%, Neutrophil percentage; CRP, C-reactive protein; CVD, Cardiovascular Disease.

Supplementary Table S2. Demographic Equivalence between Training and Test Cohorts.

Variables	Training cohort	Test cohort	*p* value
	(n = 211)	(n = 141)	
Age			0.304
<68 years	118 (55.9%)	71 (50.3%)	
≥68 years	93 (44.0%)	70 (49.6%)	
Gender			0.634
Male	174 (82.5%)	119 (84.4%)	
Female	37 (17.5%)	22 (15.6%)	
Smoking history			0.494
Smoker	87 (41.2%)	53 (37.6%)	
Never	124 (58.3)	88 (62.4%)	
GOLD stage			0.248
GOLD I-II	115 (54.5%)	68 (48.2%)	
GOLD III-IV	96 (45.5%)	73 (51.8%)	
AECOPD			0.913
Yes	102 (48.3%)	69 (48.9%)	
No	109 (51.7%)	72 (51.1%)	

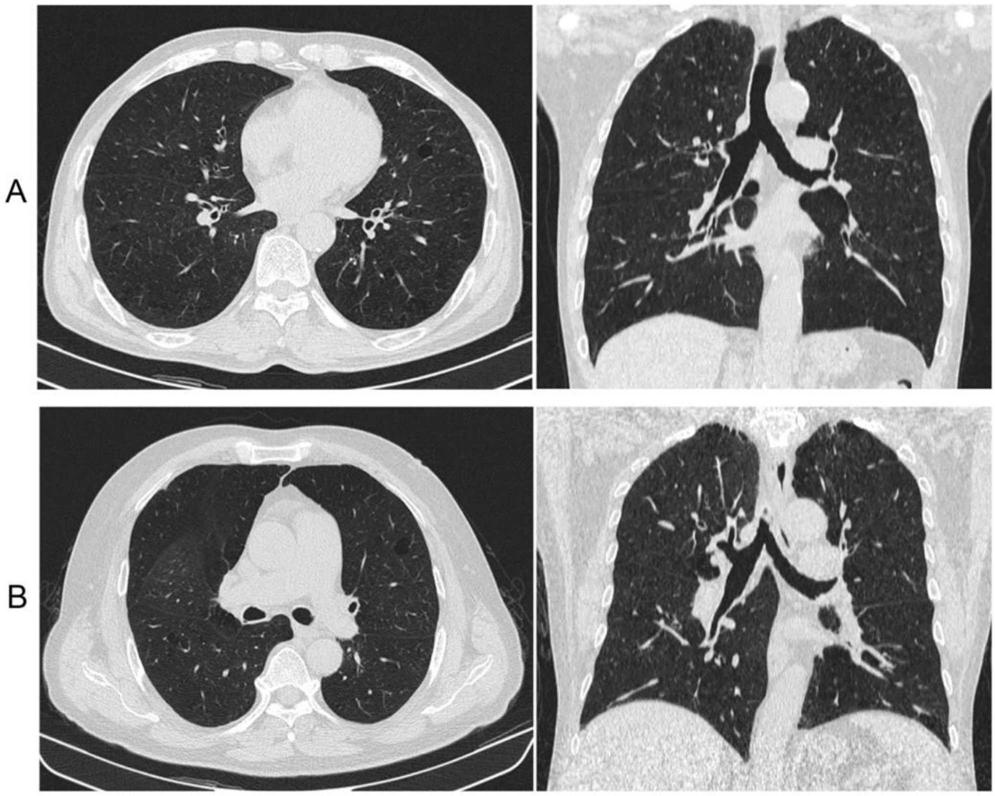

Supplementary Figure S1. Representative inspiratory chest CT images in coronal and axial planes. (A) Stable COPD (69-year-old male, GOLD II): Minimal emphysema and preserved parenchymal architecture. (B)Noninfectious AECOPD (57-year-old male, GOLD III): Diffuse emphysema and bronchial wall thickening.

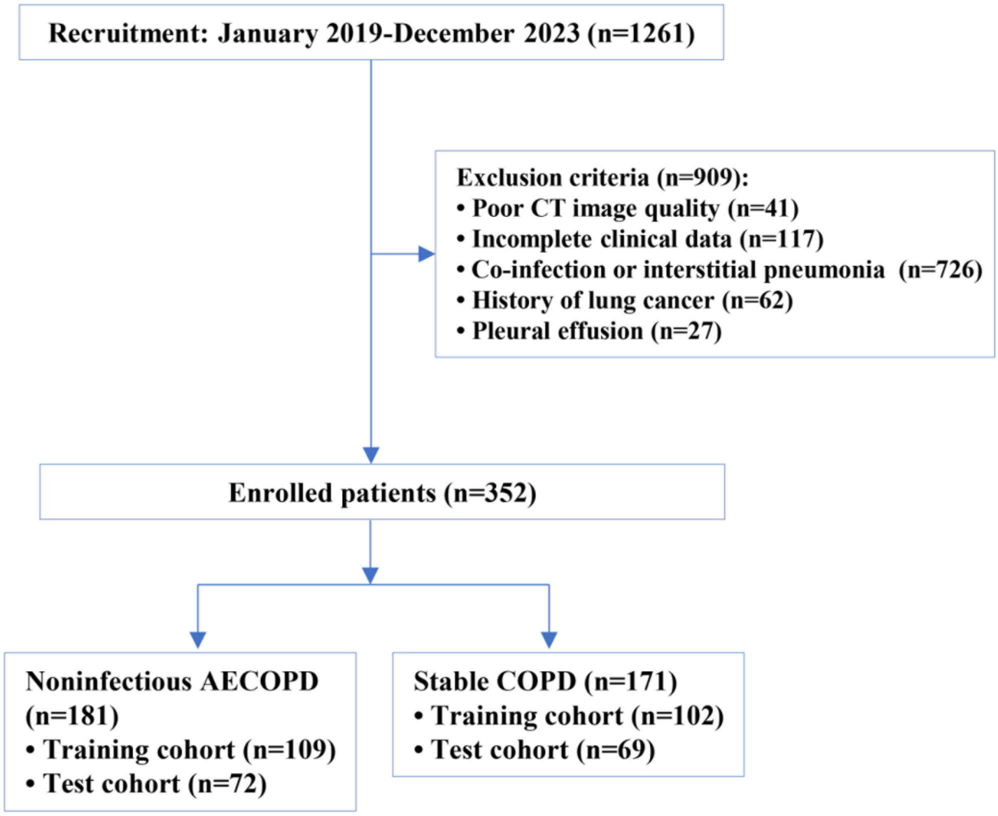

Supplementary Figure S2. Patient selection flowchart.

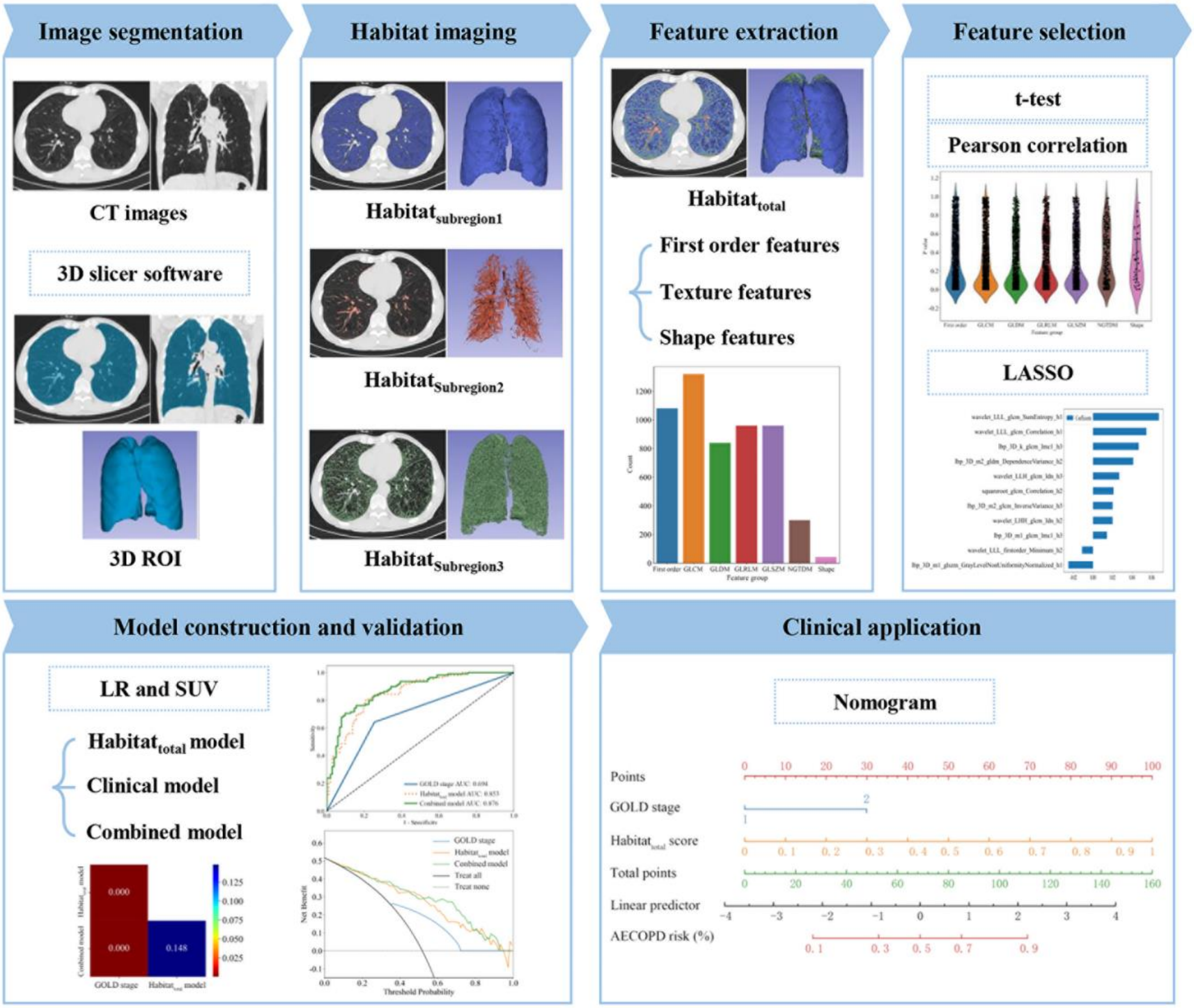

Supplementary Figure S3. Comprehensive Workflow for Whole-Lung Habitat Radiomics Analysis. Comprehensive workflow encompassing CT acquisition, lung segmentation, voxel-wise habitat mapping, radiomic feature extraction, machine learning modeling, and clinical integration.

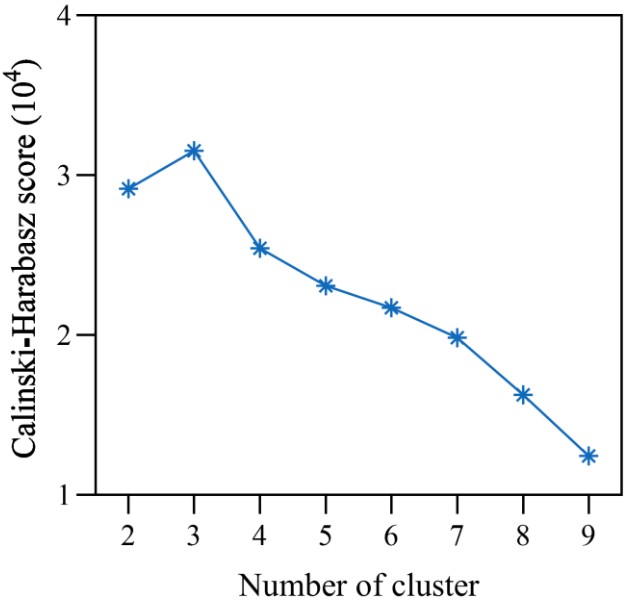

Supplementary Figure S4. Cluster number optimization using the Calinski-Harabasz score. Maximum index score (3.15×10^4) at k=3 confirms optimal habitat partitioning into three distinct subregions.

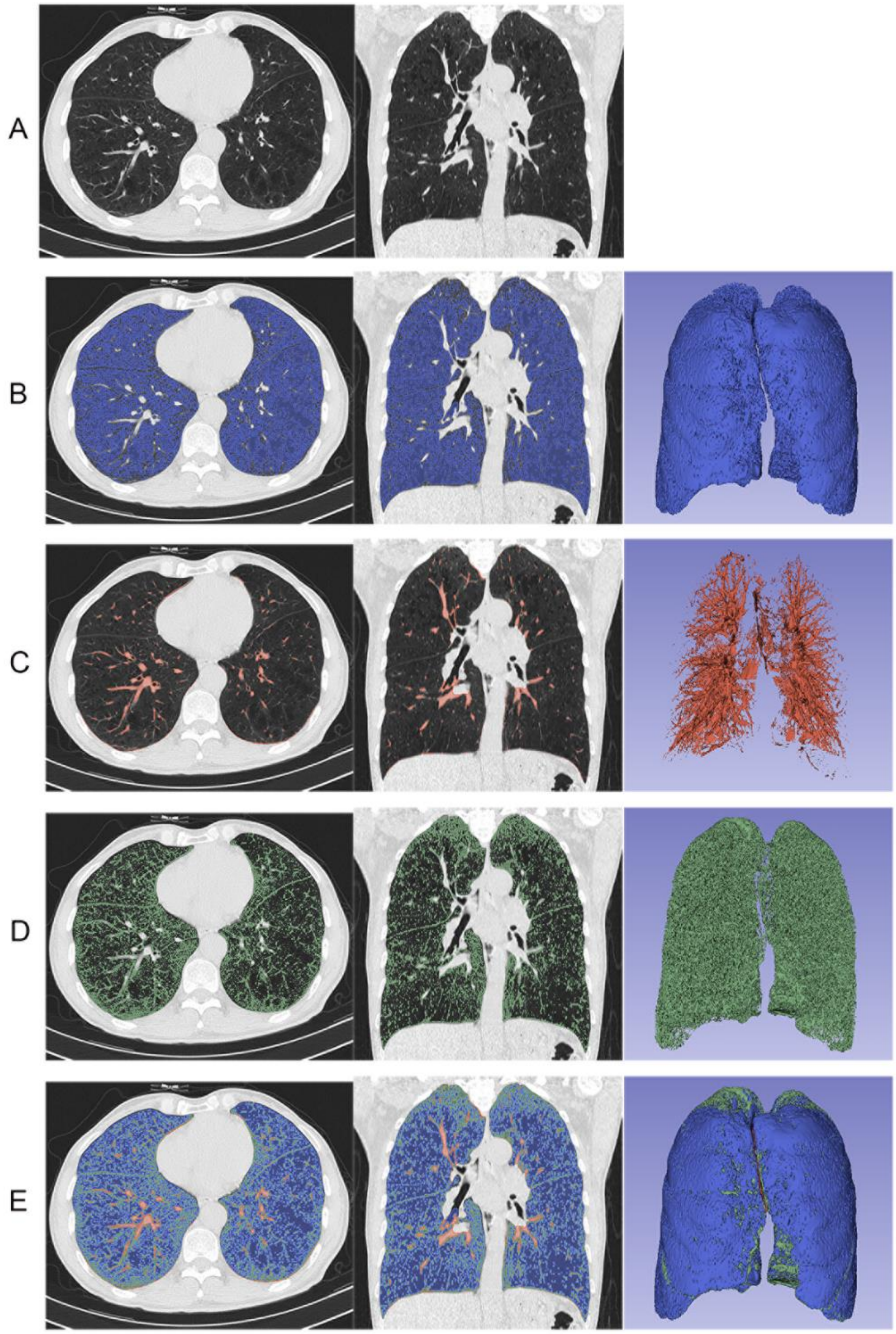

Supplementary Figure S5. Whole-lung habitat mapping in a representative noninfectious AECOPD patient. (A) Baseline inspiratory CT. (B) Subregion 1 (predominantly corresponded to emphysema/bullae, blue). (C) Subregion 2 (predominantly corresponded to bronchovascular bundles, red). (D) Subregion 3 (predominantly corresponded to parenchyma, green). (E) Integrated habitat map.

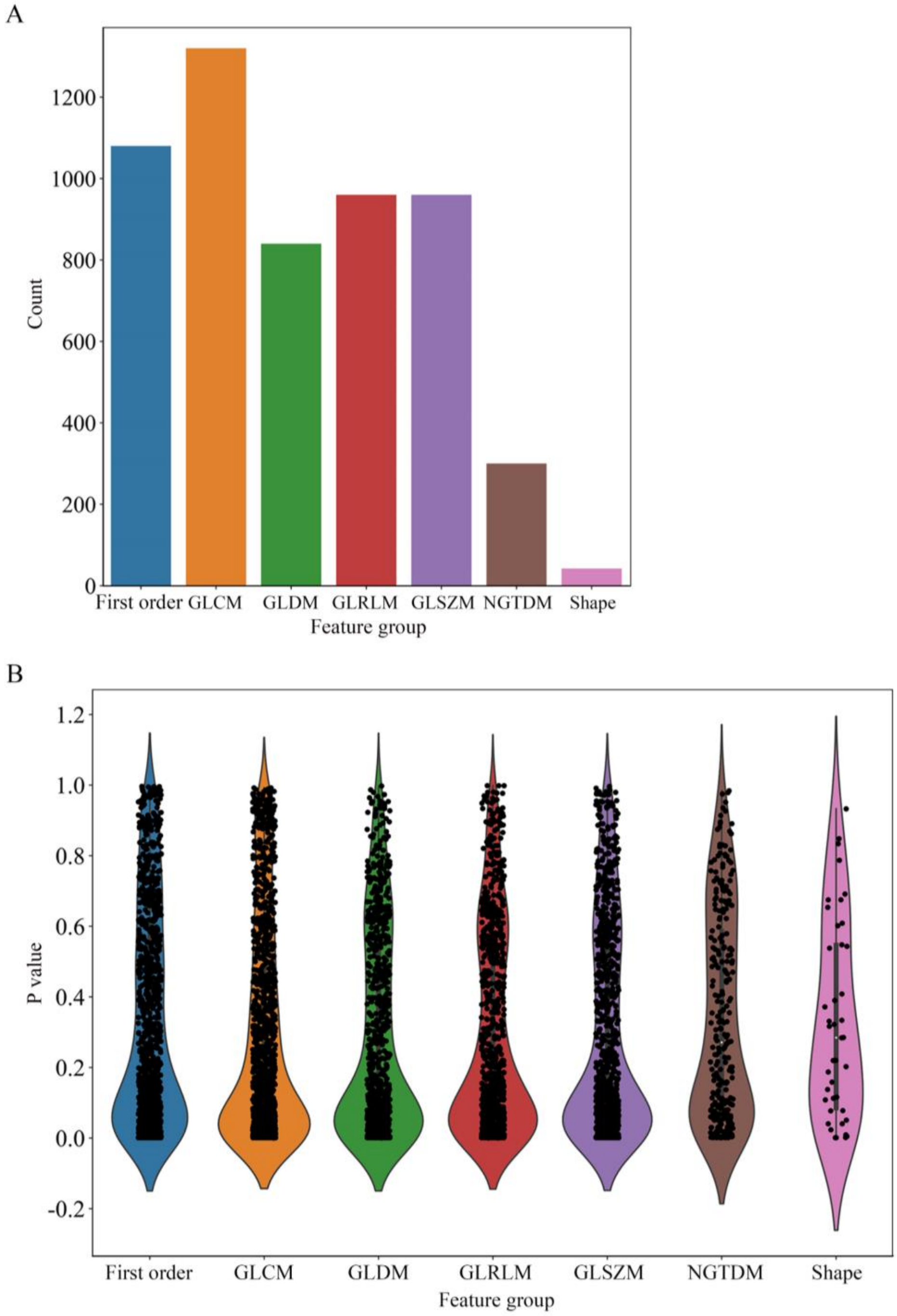

Supplementary Figure S6. Radiomic feature distribution(A) and selection(B).

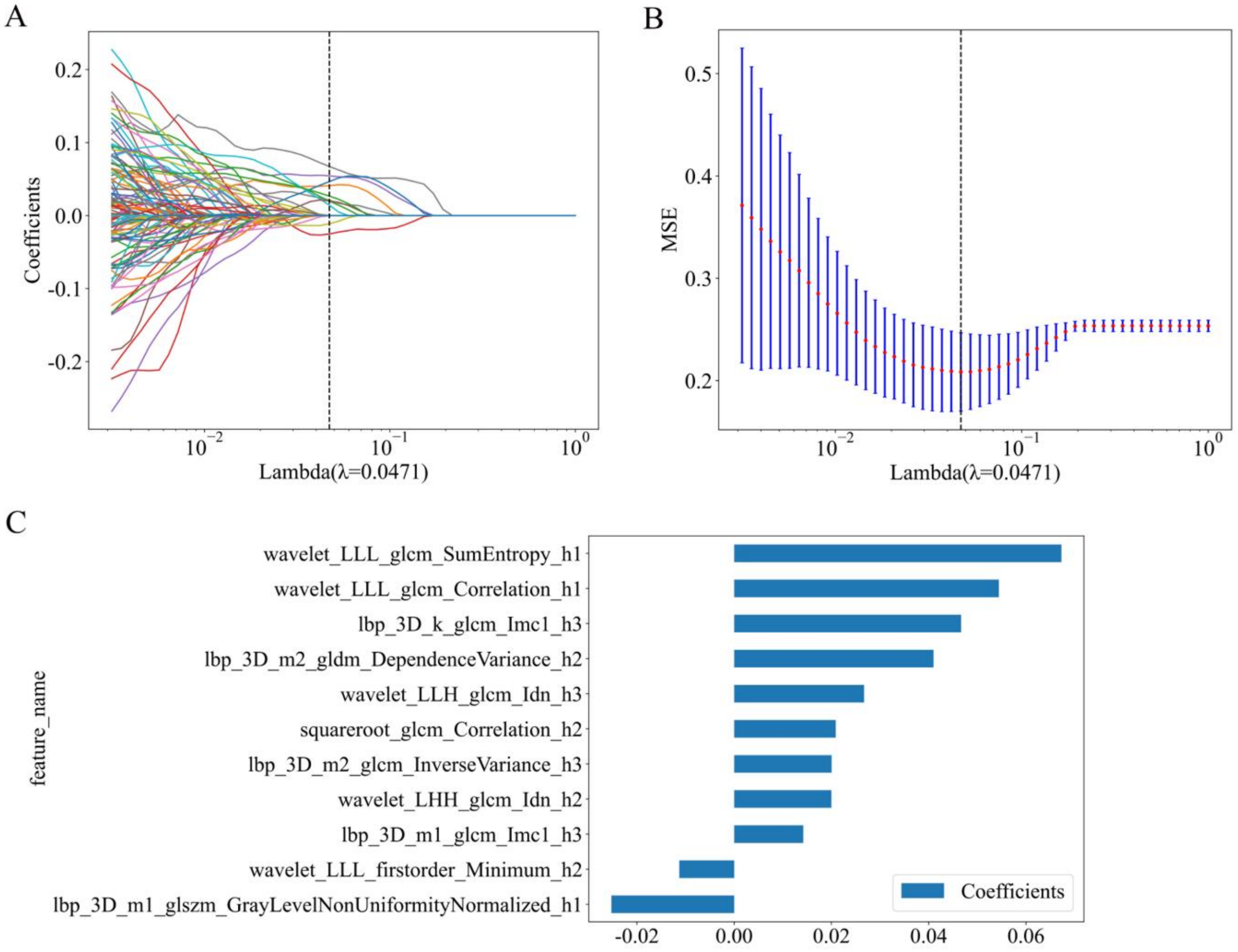

Supplementary Figure S7. Feature selection via LASSO regression for the whole-lung habitat model. (A) Coefficient trajectories across regularization parameters. (B) Optimal λ selection (λ=0.082) using 10-fold cross-validation. (B) Eleven radiomic features retained with non-zero coefficients.

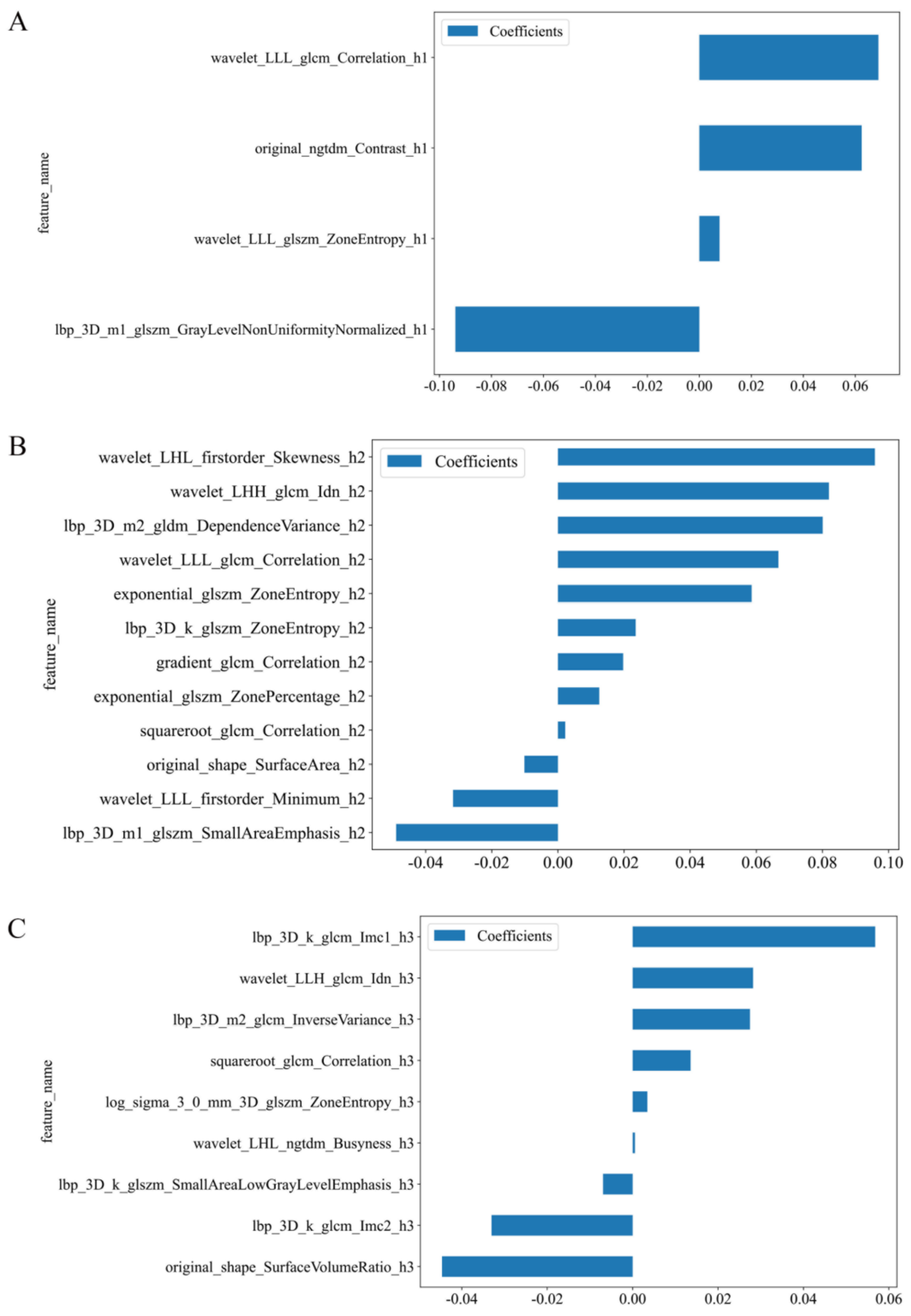

Supplementary Figure S8. LASSO feature selection for subregion-specific models. (A) Subregion 1: 4 features. (B) Subregion 2: 12 features. (C) Subregion 3: 9 features.

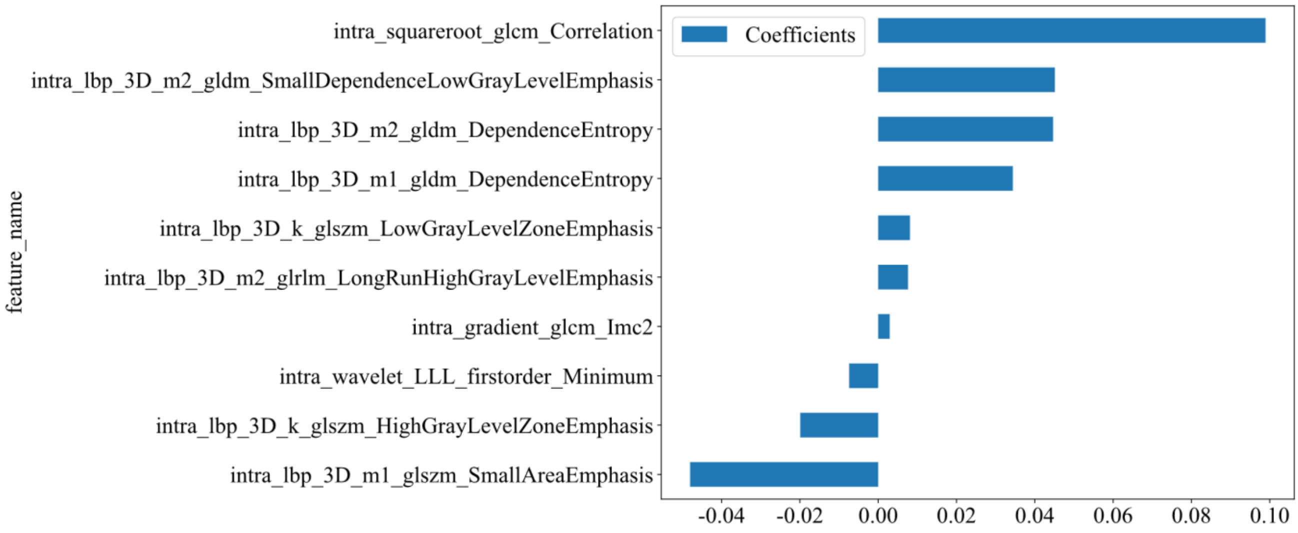

Supplementary Figure S9. 10 core features in traditional radiomics models.

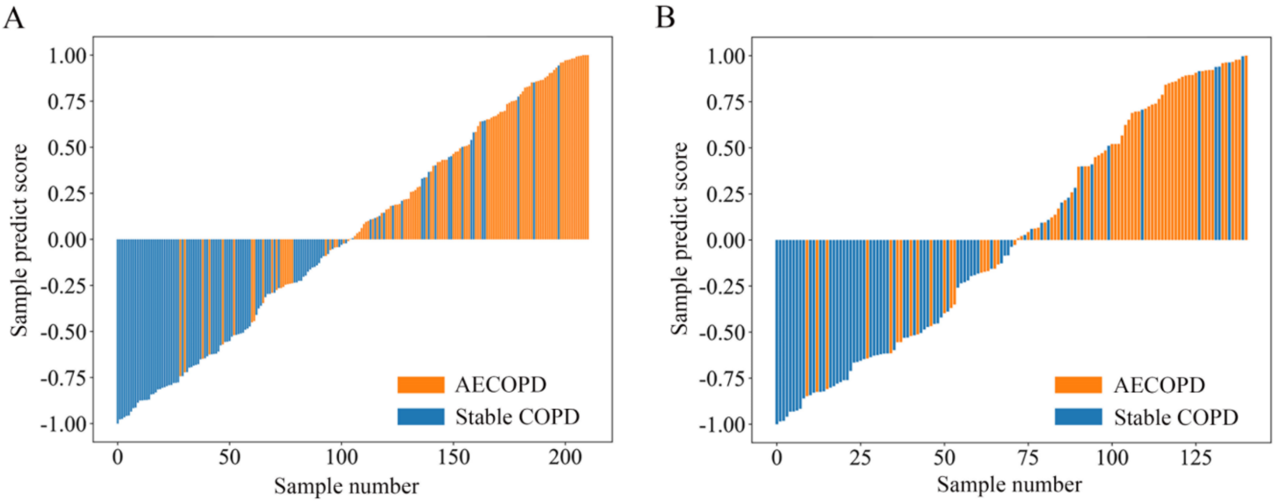

Supplementary Figure S10 Prediction probability distributions of the habitat$_{total}$ model. Density plots demonstrate progressive separation between stable COPD and noninfectious AECOPD classifications without evidence of overfitting in training (A) and test (B) cohorts.

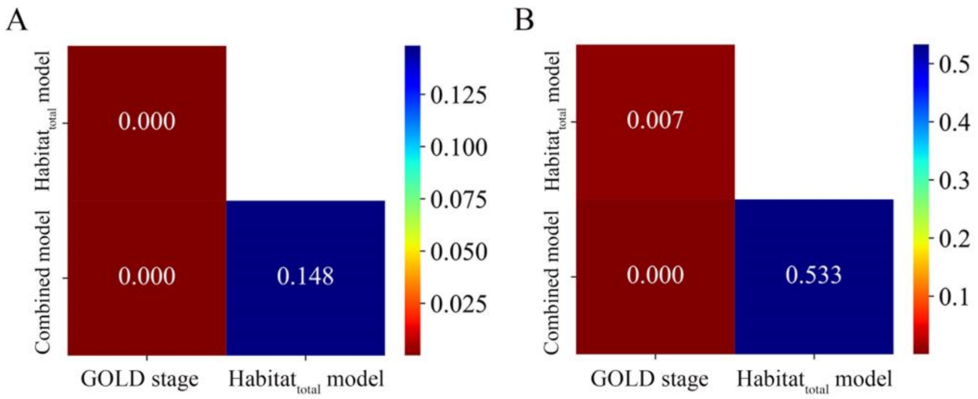

Supplementary Figure S11. DeLong's test for model comparison in the training(A) and test(B) cohorts, respectively.

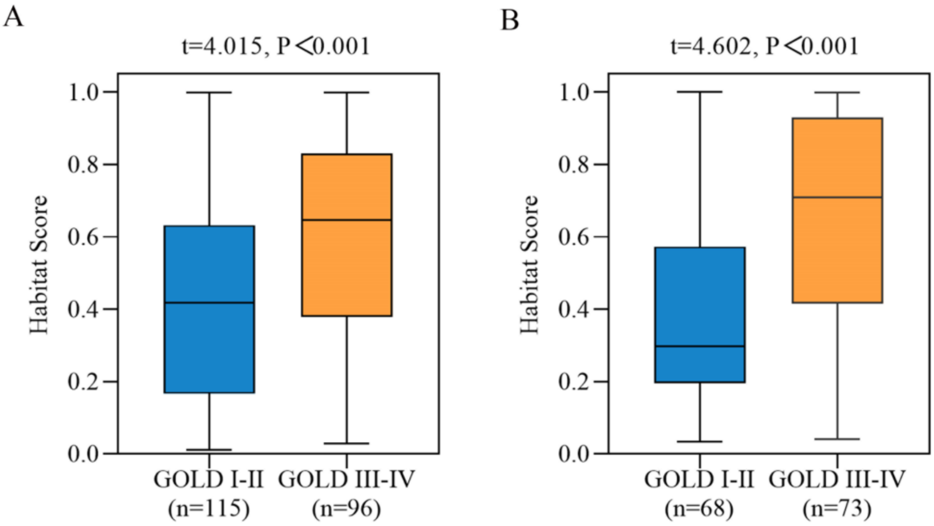

Supplementary Figure S12. Comparative Analysis of $Habitat_{total}$ Radiomic Scores and GOLD Stage Stratification in the training (A) and test (B) cohorts, respectively.
